# Autism spectrum disorders as a risk factor for adolescent self-harm: a retrospective cohort study of 113,286 young people in the UK

**DOI:** 10.1186/s12916-022-02329-w

**Published:** 2022-04-29

**Authors:** Emily Widnall, Sophie Epstein, Catherine Polling, Sumithra Velupillai, Amelia Jewell, Rina Dutta, Emily Simonoff, Robert Stewart, Ruth Gilbert, Tamsin Ford, Matthew Hotopf, Richard D. Hayes, Johnny Downs

**Affiliations:** 1 Institute of Psychiatry, Psychology and Neuroscience, King’s College London, London, UK; 2 Department of Population Health Sciences, University of Bristol, Bristol, UK; 3 South London and Maudsley NHS Foundation Trust, London, UK; 4 Population, Policy and Practice Research and Teaching Department, UCL Great Ormond Street Institute of Child Health, London, UK; 5 Department of Psychiatry, University of Cambridge, Cambridge, UK

**Keywords:** Child and adolescent mental health, Epidemiology, Autism spectrum disorders, Education, Data linkage

## Abstract

**Background:**

Individuals with autism spectrum disorder (ASD) are at particularly high risk of suicide and suicide attempts. Presentation to a hospital with self-harm is one of the strongest risk factors for later suicide. We describe the use of a novel data linkage between routinely collected education data and child and adolescent mental health data to examine whether adolescents with ASD are at higher risk than the general population of presenting to emergency care with self-harm.

**Methods:**

A retrospective cohort study was conducted on the population aged 11–17 resident in four South London boroughs between January 2009 and March 2013, attending state secondary schools, identified in the National Pupil Database (NPD). Exposure data on ASD status were derived from the NPD. We used Cox regression to model time to first self-harm presentation to the Emergency Department (ED).

**Results:**

One thousand twenty adolescents presented to the ED with self-harm, and 763 matched to the NPD. The sample for analysis included 113,286 adolescents (2.2% with ASD). For boys only, there was an increased risk of self-harm associated with ASD (adjusted hazard ratio 2·79, 95% CI 1·40–5·57, *P*<0·01). Several other factors including school absence, exclusion from school and having been in foster care were also associated with a higher risk of self-harm.

**Conclusions:**

This study provides evidence that ASD in boys, and other educational, social and clinical factors, are risk factors for emergency presentation with self-harm in adolescents. These findings are an important step in developing early recognition and prevention programmes.

**Supplementary Information:**

The online version contains supplementary material available at 10.1186/s12916-022-02329-w.

## Background

Self-harm is common in adolescents with approximately 18% reporting having self-harmed before the age of 18 [1]. Only one in eight adolescent self-harming episodes involve hospital presentations, [2–4] generally when the incident is too severe to be self-managed [5]. Presentations to hospitals with self-harm represent one of the strongest risk factors for future suicide [6]. Within the UK, a study of serious case reviews found 10–20% of young people who die by suicide visit a hospital for self-harm in the year prior to their death [7, 8].

The definition of self-harm used in this study is ‘any act of self-poisoning or self-injury carried out by an individual regardless of motivation’ [9]. This definition (rather than a distinction between non-suicidal self-injury and suicide attempts) is often used, particularly in adolescents, due to the mixed motivation that is often involved, the difficulties in determining intent and the fact that self-harm, regardless of the presence or absence of suicidal intent, is strongly associated with a greater risk of subsequent suicide [10, 11].

Population surveys of adolescents show prevalence of self-harm in the past year differs between genders: 11% of girls compared to 3–6% of boys [12–14]. Depression and anxiety, low self-esteem, impulsivity, attention and conduct difficulties are the most replicated risk factors for self-harm [4, 13, 15]. Victims of maltreatment, those with lower socio-economic status, those excluded from school, or those with prolonged absence from school are also potentially more at risk [16–19].

Findings emerging from recent epidemiological studies on suicidal behaviour in adulthood support the hypothesis that higher rates of self-harm could be expected in adolescents with ASD. 66% of adults newly diagnosed with ASD in a clinical sample reported that they had contemplated suicide (UK general population prevalence 17%) while 35% had planned or attempted suicide [20]. The risk of suicide attempts has also been reported as five times higher in adults with ASD compared to non-ASD controls [21].

In adolescents, from the limited research conducted, findings show those with ASD are also at a greater risk of suicidal behaviours [22]. For example, one clinical study found over one in six young people with ASD contemplated or attempted suicide during childhood, making them 30 times more at risk than typically developing children [23]. However, the practical implications of these studies are difficult to judge, as qualitatively diverse events (e.g. suicidal ideation vs. suicide attempt) have been aggregated into binary outcomes. Furthermore, the methodological approaches of these studies, including cross-sectional designs, small, selective and mostly clinical samples, as well as lack of adequate adjustment for possible confounding factors or comparable control groups, [22–25] further limit the interpretation and generalisability of these findings.

To our knowledge, there have been no population-based cohort studies which have tested the hypothesis that the risk of presentation with self-harm to emergency care is raised in young people with ASD. Furthermore, to improve long-term outcomes for adolescents and reduce risks of self-harm, robust data is needed as a reference for health and educational authorities to use for targeted policy development. Therefore, our main aim was to address limitations of previous work, test the hypothesis that ASD is associated with raised self-harm risk in adolescence and quantify the extent of the potential risk. A secondary aim was to examine the associations between self-harm and a number of other potential socio-demographic, economic, health and educational risk factors. To meet these aims, we conducted a historical cohort data linkage study using routinely collected data from school census records matched to psychiatric records. We examined population rates of self-harm outcomes by age and gender and ensured that our analysis generalised to mainstream secondary schools.

## Methods

### Study population

The inclusion criteria were adolescents aged 11–17 who were enrolled in a state-maintained school and resident in one of four South London Boroughs (Southwark, Lewisham, Lambeth and Croydon). No exclusion criteria were applied based on diagnosis or any other individual characteristics. Individuals were followed-up from their 11th birthday or on 1 April 2009, whichever was later, until first presentation to the Emergency Department (ED) with an act of self-harm, their 18th birthday or on 31 March 2013, whichever occurred sooner.

### Sources of data

Data were derived from the National Pupil Database (NPD), an anonymised dataset managed by the Department for Education (DfE). The DfE provided whole region individual level data for the four boroughs. Pupils’ data were linked to electronic child and adolescent mental health records within the Clinical Record Interactive Search (CRIS) [26, 27] system for any individuals who had a history of contact with South London and Maudsley NHS Foundation Trust (SLaM) [28]. CRIS comprises data derived from the electronic health record system (ePJS) used to record all clinical contacts within SLaM. Data were linked by the DfE by matching personal identifiers (name, date of birth and postcode) using fuzzy and deterministic approaches, under robust governance protocols [28]. CRIS data were separately linked to Hospital Episodes Statistics (HES), which provides information on all ED attendances (HES Accident and Emergency (A&E) data) and admissions (HES Admitted Patient Care (APC) data) to National Health Service (NHS) hospitals. This study does not include any data linkage between HES and NPD.

### Outcome data

The primary outcome was first attendance to acute hospital ED with self-harm as defined by the National Institute for Health and Care Excellence (NICE) [29] as interpreted and recorded by the assessing mental health professional. Cases described as self-injurious behaviour typically associated with intellectual disability and developmental disorders were excluded.

During the study period, SLaM provided psychiatric liaison services within the local catchment’s four main NHS EDs. The EDs refer all attendees with self-harm for a SLaM psychiatric assessment, and liaison teams record all referrals using ePJS (therefore data available within CRIS) [30]. Data on self-harm were ascertained from free-text records within CRIS using methods described in a previous ED self-harm study involving identifying the date and time of any ED attendances using HES [30].

ICD-10 diagnosis data were extracted from CRIS to provide further descriptive characteristics of adolescents who had attended ED with self-harm. Low frequency psychiatric diagnoses were collapsed into a single category labelled ‘Other’.

### Exposure data

The NPD special education needs (SEN) register was used to identify all ASD diagnoses and other SEN including learning difficulties, behavioural, emotional and social (BES) problems, speech, language or communication needs or hearing, vision or physical disabilities. The ASD SEN category was only applied if a formal diagnosis of ASD had been made by health professionals, either within mental health or paediatric health services, according to the UK government SEN code of practice [31]. To reduce the potential for reverse causality between self-harm and BES problems (i.e. where self-harm or associated mental health difficulties are the reason for assigning this category of need), this SEN category was only coded before the start of follow-up for each individual. An individual can be assigned up to two SEN categories, primary and secondary. Attention deficit hyperactivity disorder (ADHD or hyperkinetic disorder) was not classified as a SEN category in the NPD, but is a highly prevalent neurodevelopmental disorder and important to characterise alongside ASD and learning disabilities. Diagnoses of ADHD captured within CRIS provided a good approximation of population prevalence, given that almost all ADHD diagnostic services within the four boroughs were provided by SLaM. Therefore, any diagnoses of ADHD were extracted from CRIS using methodology described in previous studies [32–34]. It has been longstanding diagnostic practice in UK clinics that ASD is recognised as co-occurring with other neurodevelopmental and mental health conditions, including ADHD, a practice now codified in ICD-11 and DSM-5 and diagnostic guidance. A review of co-morbidity patterns in children with ASD from the same clinical catchment area as the study sample, found over 54% received an additional psychiatric diagnosis, with the majority coded with co-occurring ADHD [32]*.*

NPD census data were used to provide details on gender, ethnicity and English as a second language (via parental report) with missing data replaced by linked health data. Additional baseline characteristics including socio-economic status using free school meal eligibility (based on means testing) as a proxy as well as neighbourhood deprivation (by home address) [35] and whether children were under care of the local authority were extracted from the NPD for the academic year prior to start of follow-up. Summer birth (May-Aug) was derived from date of birth. This variable was included due to evidence that individuals who are the youngest in the school year are at increased risk of a range of mental health problems [36, 37].

Educational attainment data was retrieved from Key Stage 2 (KS2) Standardised Attainment Tests (SATs). SATs are school assessments that measure children’s educational achievement and are taken by children aged 10–11. Ranked *z*-scores were created from average total test marks across English, Maths and Science. This ranked score was then divided into five quintiles. SAT data was missing for pupils with a KS2 level ‘B’ (working below test levels); anyone assigned ‘B’ is therefore within the lowest quintile. Binary outcome markers were created for poor attendance (< 80%) for the academic year before start of follow-up and for pupils with a prior record of exclusion (fixed term or permanent) up to the point of study entry.

### Statistical analysis

Due to established differential risks for self-harm according to gender [38], all analyses were conducted separately for girls and boys. Statistical disclosure rules (a condition of NPD and CRIS use) required us not to publish counts of less than ten. Exact numbers for certain groups are therefore not presented. Using NPD data to provide the regional population denominator, incidents of self-harm following study entry were derived, by gender and each year of ages 11–17. Having checked proportional hazards assumptions, potential risk factors for hospital presentation with self-harm were assessed separately in unadjusted Cox regression analyses and then together in an adjusted model. Baseline (time zero) was set at the date of 11th birthday or 1 April 2009 (whichever was later), and data was censored at first ED presentation with self-harm, 18th birthday or 31 March 2013 (whichever occurred sooner). Sensitivity analyses included a restricted sample of pupils attending only mainstream secondary schools (as special schools or pupil referral units are likely to have populations with greater psychiatric morbidity) and pupils who entered the study at age 11. Multiple imputation was conducted as a sensitivity analysis to examine whether missing data, including that related to non-matching between the NPD and CRIS, caused substantial changes in the effect estimate of any association between ASD and self-harm. Data were assumed missing at random due to availability of complete outcome data and a considerable number of predictor variables relating to non-linkage [39]. Following recent recommendations [40], ten imputed data sets were created (*m*=10). All analyses were completed in STATA version 14·0.

## Results

**Fig. 1:**
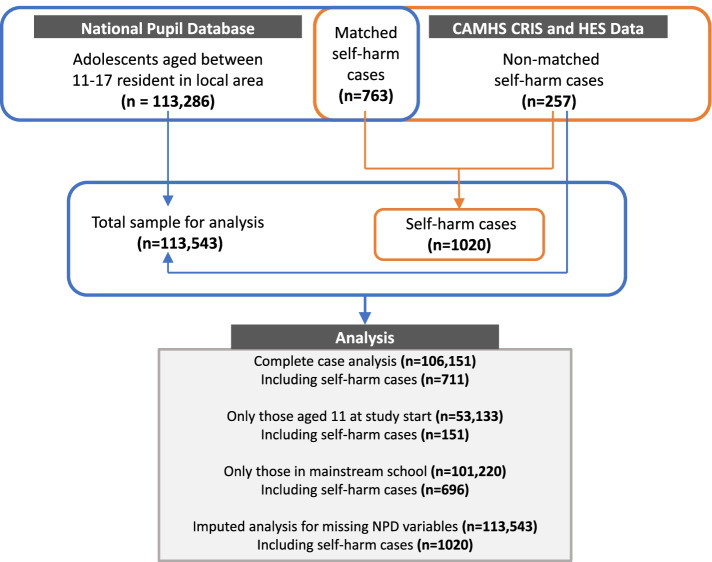
Data sources, linkage and sample sizes for complete case analysis and sensitivity analyses

Figure 1 shows how the adolescent population sample, and self-harm cases were ascertained. Using census data from the NPD for the four boroughs served by SLaM, 113,286 adolescents were eligible for study entry. Mean follow-up was 2·71 years (SD 1·25, range 0·3–3·99). Total follow-up time was 308,246·2 person-years. During follow-up, 1020 adolescents (0.9%) attended ED or were admitted to hospital with at least one episode of self-harm. Of these, 763 adolescents (75%) were successfully matched to the NPD and therefore included in the main analysis, providing a self-harm incidence rate of 33 ED self-harm events per 10 000 persons/year (95% CI 31–35). An additional 257 cases were identified (from CRIS self-harm data and HES APC data) who were not matched to the NPD resulting in a total sample of 113,543 after imputation of missing data (conducted as a sensitivity analysis).

### Characteristics of adolescents presenting with self-harm

Of the 1020 adolescents (834 girls, 186 boys) presenting with self-harm, the mean age of the first presentation was 15·9 and 15·6 years for boys and girls, respectively (Table 1). At the time of self-harm presentation, fewer than 50% had prior history of contact with SLaM. The most common reason for presentation was self-poisoning/overdose (boys 51%, girls 74%), followed by cutting and other forms of self-injury. In terms of ICD-10 disorders, the most prevalent were depressive disorders (boys 29%, girls 33%), anxiety (22%) and childhood onset emotional and behavioural disorders (boys 18%, girls 15%). Multiple diagnoses for one individual were possible.

**Table 1:** Characteristics of 1020 adolescents aged 11–17 living in the SLaM catchment area presenting to the 4 local EDs with self-harm between March 2009 and March 2013

Characteristics	Self-harm presentations (***n***, %)	
	Male (*n*=186)	Female (*n*=834)
Mean age at first self-harm presentation (SD)	15.9 (1.9)	15.6 (1.4)
Known to MH services prior to self-harm	83 (44.6)	407 (48.8)
**Ethnicity**		
White	88 (47.3)	357 (42.8)
Black	28 (15.0)	212 (25.4)
Asian	12 (6.5)	47 (5.7)
Mixed	16 (8.6)	102 (12.2)
Other	12 (6.5)	29 (3.5)
Not disclosed/unknown	30 (16.1)	87 (10.4)
**National neighbourhood deprivation**		
Most deprived quintile	63 (33.9)	320 (38.4)
2nd	79 (42.5)	330 (39.5)
3rd	32 (17.2)	120 (14.4)
4th	<10 (<5.4)	44 (5.3)
Least deprived quintile	<10 (<5.4)	20 (2.4)
**Type of self-harm**		
Self-poisoning or overdose	95 (51.1)	617 (74.0)
Self-injury (cutting, stabbing, self-battery)	74 (39.8)	171 (20.5)
Both self-poisoning and self-injury	<10 (<5.4)	29 (3.5)
Other (hanging, jumping from a height, immersion in water)	14 (7.5)	17 (2.0)
**ICD-10 Axis 1**^b^**(pre- or post-first self-harm)**	**No. and prevalence of disorders (%)**^a^	
Substance misuse disorders (F10–19)	10 (5.4)	13 (1.5)
Depressive disorder (F32)	53 (28.5)	277 (33.2)
Psychotic disorders (F20–29,31,32·3, F33·3)	<10 (<5.4)	<10 (<1.2)
Anxiety disorder (F40–42, F43–F48)	42 (22.5)	186 (22.3)
Eating disorder	<10 (<5.4)	17 (2.0)
Autism spectrum disorders (F84)	18 (9.7)	21 (2.5)
Hyperkinetic disorder (F90)	19 (10.2)	15 (1.7)
Child-onset emotional and behavioural disorders (F91-F98)	33 (17.7)	127 (15.2)
No diagnosis	41 (22.0)	249 (29.9)
Other	<10 (<5.4)	21 (2.5)
**Axis 3 intellectual disability**	<10 (<5.4)	<10 (<1.2)

^a^Multiple morbidities could be counted for each individual

^b^Axis I: Clinical syndromes (psychiatric disorders including personality disorders and somatic diseases)

### Characteristics of the whole sample and subsample with ASD

Table 2 provides a breakdown of characteristics of the sample, by gender and ASD status, provided by NPD (omitting non-matched self-harm data, *n*=257). There was considerable ethnic, socio-economic and cultural diversity within the sample, with non-white ethnic groups making up approximately two thirds of the study population and over 25% reporting English as their second language. Approximately, 80% of the sample resided in neighbourhoods within the highest 40% for national deprivation, with over 25% of adolescents from families eligible for free school meals [41].

**Table 2:** Socio-demographic, educational and clinical characteristics of the population resident in South London by gender and ASD status

Sample characteristics*	Male (*n*=56,578)		Female (*n*=56,708)	
	No ASD (*n*=54,552)	ASD (*n*=2026)	No ASD (*n*=56,271)	ASD (*n*=437)
**Self-harm presentation to ED**	107 (0.2)	11 (0.5)	635–645^**^ (1.1–1.1)	<10 (<2·3)
Mean age at baseline (SD)	12.8 (2.0)	12.3 (1.7)	12.8 (2.0)	12.2 (1.6)
Mean duration of follow-up (SD)	2.74 (1.3)	2.73 (1.3)	2.72 (1.3)	2.80 (1.3)
**Ethnicity**	(*n*, %)	(*n*, %)	(*n*, %)	(*n*, %)
White	20,238 (37.1)	770 (38.0)	20,651 (36.7)	176 (40.3)
Black	20, 012 (36.7)	850 (42.0)	21,099 (37.5)	174 (39.8)
Asian	4788 (8.8)	78 (3.9)	4869 (8.7)	24 (5.5)
Mixed	6013 (11.0)	237 (11.7)	6275 (11.2)	45 (10.3)
Other	1928 (3.5)	42 (2.1)	1891 (3.4)	<10 (<2.3)
Not disclosed/unknown	1575 (2.9)	49 (2.4)	1486 (2.6)	12 (2.8)
**National neighbourhood deprivation**				
Most deprived quintile	19,805 (36.3)	824 (40.8)	20,222 (40.0)	173 (39.6)
2nd	22,100 (40.5)	804 (39.8)	22,794 (40.5)	179 (40.1)
3rd	7759 (14.2)	251 (12.4)	8227 (14.6)	550 (12.6)
4th	3283 (6.0)	99 (4.9)	3322 (5.9)	24 (5.5)
Least deprived quintile	1579 (2.9)	42 (2.1)	1688 (3.0)	<10 (<2.3)
**Special education needs**^a^				
Learning Difficulties (specific/moderate)	8898 (16.3)	548 (27.1)	6085 (10.8)	133 (30.4)
Learning Difficulties (severe/ profound)	591 (1.1)	250 (12.3)	381 (0.7)	65 (14.9)
Behavioural, Emotional, Social problems	6726 (12.3)	548 (27.1)	3548 (6.3)	89 (20.4)
Speech, language and communication	4291 (7.9)	806 (39.8)	2134 (3.8)	161 (36.8)
Hearing, vision or physical disability	795 (1.5)	69 (3.4)	735 (1.3)	16 (3.4)
**First language**^a^				
English	39,920 (73.2)	1661 (82.0)	40,815 (72.5)	344 (78.7)
Other	13,612 (25.0)	341 (16.8)	14, 541 (25.9)	89 (20.4)
Not disclosed	1022 (1.9)	24 (1.2)	915 (1.6)	<10 (<2.3)
**School type**				
Mainstream	53,868 (98.7)	1597 (78.8)	56,024 (99.6)	333 (76.2)
Special school	579 (1.1)	418 (20.6)	237 (0.4)	104 (23.8)
Pupil referral units	107 (0.2)	11 (0.5)	10 (0.02)	0 (0)
**Educational attainment (key stage two)**^b^				
Lowest quintile	12, 220 (23.1)	1146 (59.0)	10, 461 (19.2)	296 (69.7)
Second	10,461 (19.8)	277 (14.3)	10,750 (19.7)	55 (12.9)
Third	10,301(19.5)	224 (11.5)	11,141 (20.4)	29 (6.8)
Fourth	10,283 (19.5)	168 (8.6)	10,078 (20.3)	22 (5.2)
Highest quintile	9577 (18.1)	128 (6.6)	11,172 (20.4)	23 (5.4)
**Less than 80% attendance**^c^	2587 (4.9)	118 (6.0)	2538 (4.7)	22 (5.2)
**Fixed term exclusions**^a^	5847 (10.7)	239 (11.8)	2790 (5.0)	26 (6.0)
**Other social factors**				
Summer birth (May-Aug)	18,941 (34.7)	720 (35.5)	19,185 (34.1)	140 (32.0)
Free school meals ^a^	13,105 (24.0)	696 (34.5)	13,391 (23.8)	167 (38.2)
Looked after child status ^d^	420 (0.8)	30 (1.5)	397 (0.7)	12 (2.8)
**ICD-10 hyperkinetic disorder**	670 (1.2)	131 (6.5)	168 (0.3)	23 (5.3)

^*^Missing = 257 non-matched self-harm cases

^**^Range presented to avoid statistical disclosure in the ASD group

Missing values. ^a^257, ^b^3731, ^c^4049, and ^d^4547

There were 2463 (2·2%) adolescents with ASD recorded as a SEN in the NPD. The majority of adolescents with ASD were being taught within mainstream schools (>75%), but 59–70% were in the lowest 20% for KS2 attainment. Twelve to 15% were recognised as having severe or profound learning difficulties (0.7–1.1% in the non-ASD group). For the ASD group, the mean age was 12 at study entry, with similar length of follow-up (mean 2.7 years). Around 12% of boys and 6% of girls with ASD had received at least one exclusion. Between 5 and 6% did not attend school for more than 80% of available lessons in the year before study entry. Approximately, 7% of boys and 5% girls with ASD had co-morbid hyperkinetic disorder recorded in CRIS.

### Self-harm incidence rates by age

**Fig. 2:**
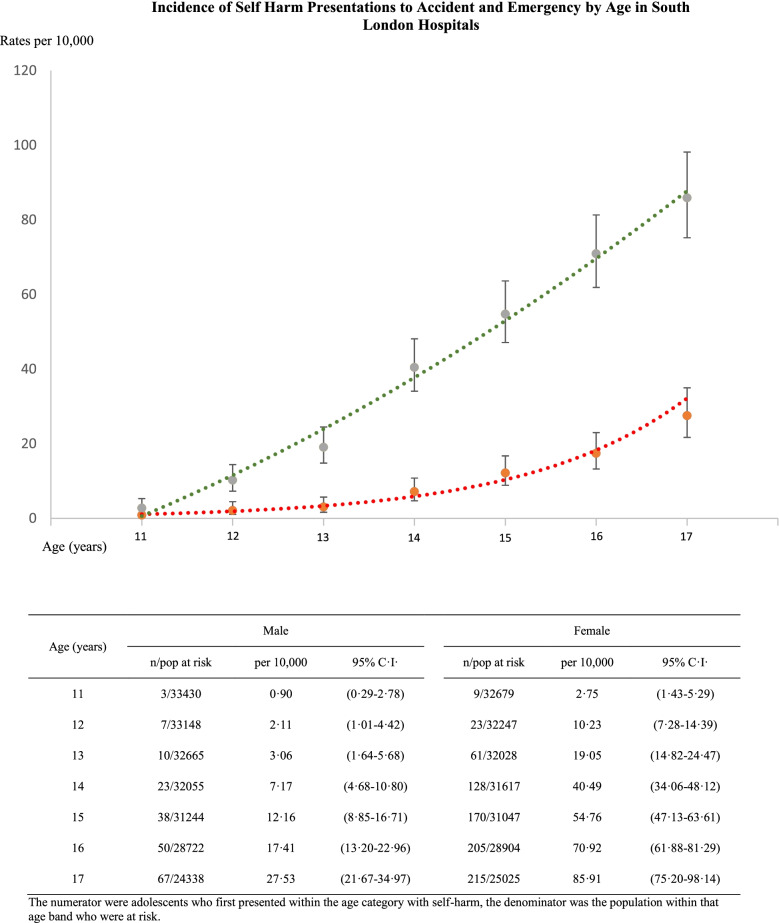
Self-harm incidence rates of adolescents presenting to ED according to age and gender, with 95% confidence intervals

Both genders show low incidence rates of self-harm presentation to ED at age 11, with a substantial increase in incidence of self-harm throughout later adolescence (Fig. 2). Incidence rates increased more than two-fold for girls from age 14 to 17 and nearly four-fold for boys.

### Association between ASD and self-harm

Tables 3 and 4 present the results of unadjusted Cox regression models for the association between ASD and self-harm stratified by gender and models adjusted for a range of social, educational and clinical risk factors/confounders.

**Table 3:** An analysis of socio-demographic risk factors for emergency presentations with self-harm amongst 113,543 adolescents residing in South London using crude and multivariable cox-regression analyses

Socio-demographic characteristics	Male (*n*=56,648)				Female (*n*=56,897)			
	No self-harm (*n*=56,462)	Self-harm (*n*=186)	Unadjusted hazard ratio	Adjusted hazard ratio^b^	No self-harm (*n*=56,063)	Self-harm (*n*=834)	Unadjusted hazard ratio	Adjusted hazard ratio^b^
Mean age at baseline (SD)	12.8 (2.1)	14.1 (1.8)	1.70 (1.55–1.86)**	1.38 (1.22–1.57)**	12.8 (2.0)	13.9 (1.8)	1.48 (1.42–1.54)**	1.28 (1.21–1.35)**
Mean duration of follow-up (SD)	2.73 (1.3)	1.89 (1.2)	-	-	2.70 (1.3)	1.86 (1.1)	-	-
**Ethnicity**	(*n*, %)	(*n*, %)			(*n*, %)	(*n*, %)		
White	20,943 (37.1)	88 (47.3)	*Reference*	*Reference*	20,534 (36.6)	357 (42.8)	*Reference*	*Reference*
Black	20,842 (36.9)	28 (15.0)	0.32 (0.21–0.48)**	0.38 (0.23–0.65)**	21,106 (37.7)	212 (25.4)	0.57 (0.48–0.68)**	0.58 (0.78–0.71)**
Asian	4860 (8.6)	12 (6.5)	0.60 (0.33–1.10)	0.87 (0.35–2.14)	4865 (8.7)	47 (5.7)	0.58 (0.43–0.78)**	0.61 (0.40–0.94)*
Mixed	6234 (11.0)	16 (8.6)	0.62 (0.36–1.04)	0.69 (0.37–1.26)	6218 (11.1)	102 (12.2)	0.97 (0.77–1.20)	1.12 (0.88–1.41)
Other	1968 (3.5)	12 (6.5)	1.42 (0.78–2.60)	0.64 (0.14–2.69)	1880 (3.3)	29 (3.5)	0.88 (0.60–1.28)	0.78 (0.46–1.31)
Not disclosed	1615 (2.9)	30 (16.1)	4.9 (3.3–7.5)**	0.74 (0.17–3.04)	1460 (2.6)	87 (10.4)	4.0 (3.16–5.04)**	0.94 (0.54–1.61)
**National neighbourhood deprivation**^a^								
Most deprived quintile	20,586 (36.5)	63 (33.9)	*Reference*	*Reference*	20,144 (35.9)	320 (38.4)	*Reference*	*Reference*
2nd	22, 855 (40.5)	78 (42.5)	1.14 (0.82–1.58)	0.98 (0.63–1.53)	22,720 (40.6)	330 (39.5)	0.92 (0.79–1.08)	0.98 (0.81–1.17)
3rd	7989 (14.2)	32 (17.2)	1.31 (0.86–2.20)	1.40 (0.81–2.42)	8193 (14.6)	120 (14.4)	0.95 (0.77–1.17)	0.88 (0.67–1.15)
4th	3378 (6.0)	<10 (<5.4)	0.78 (0.38–1.64)	0.74 (0.28–1.88)	3311 (5.9)	44 (5.3)	0.85 (0.62–1.16)	0.80 (0.55–1.18)
Least deprived	1620 (2.9)	<10 (<5.4)	0.81 (0.29–2.21)	0.27 (0.04–2.01)	1677 (3.0)	20 (2.4)	0.75 (0.48–1.18)	0.79 (0.46–1.3)

^*^P⩽0·05, ^**^P⩽0·01; ^a^missing values = 52. ^b^Adjusted for all other factors listed in Tables 3 and 4

**Table 4:** An analysis of educational and clinical risk factors for emergency presentations with self-harm (*n*=1020) amongst 113,543 adolescents aged 11–17 residing in South London using crude and multivariable cox-regression analyses

Educational and clinical characteristics	Male (*n*=56,581)				Female (*n*=56,709)			
	No self-harm (*n*=56,460)	Self-harm (*n*=120)	Unadjusted hazard ratio	Adjusted hazard ratio^e^	No self-harm (*n*=56,063)	Self-harm (*n*=646)	Unadjusted hazard ratio	Adjusted hazard ratio^e^
	(*n*, %)	(*n*, %)			(*n*, %)	(*n*, %)		
**Special education needs**^a^								
Autism spectrum disorders	2015 (3.5)	11 (9.2)	2.73 (1.47–5.09)**	2.79 (1.40–5.57)**	434 (0.8)	<10 (<1·5)	0.57 (0.18–1.78)	0.52 (0.16–1.63)
Learning difficulties (specific/moderate)	9418 (16.7)	28 (23.3)	1.44 (0.95–2.20)	1.07 (0.62–1.76)	6113 (10.9)	105 (16·3)	1.50 (1.22–1.85)**	0.99 (0.77–1.27)
Learning difficulties (severe/profound)	840 (1.5)	<10 (<8.3)	0.55 (0.08–3.92)	0.39 (0.05–2.98)	444 (0.8)	<10 (<1·5)	0.38 (0.09–1.52)	0.40 (0.10–1·67)
Behavioural, emotional, social	7235 (12.8)	39 (32.5)	3.14 (2.19–4.70)**	1.66 (1.02–2.73)*	3494 (6.2)	143 (22·1)	4.20 (3.48–5.05)**	2.31 (1.84–2.88)**
Speech, language and communication	5086 (9.0)	11 (9.2)	1.06 (0.57–1.98)	0.99 (0.51–1.95)	2269 (4.1)	26 (4·0)	1.01 (0.68–1.50)	1.13 (0.74–1.72)
Hearing, vision or physical disability	860 (1.5)	<10 (<8.3)	2.17 (0.80–5.89)	2.13 (0.77–5.85)	746 (1.3)	5 (0·8)	0.56 (0.23–1.34)	0.59 (0.25–1.42)
**First language**^a^								
English	41,482 (73.5)	100 (83.3)	*Reference*	*Reference*	40,652 (72.5)	508 (78.6)	*Reference*	*Reference*
Other	13,942 (24.7)	11 (9.2)	0.33 (0.18–0.62)**	0.50 (0.25–0.98)*	14, 529 (25.9)	101 (15.6)	0.57 (0.46–0.70)**	0.77 (0.61–0.98)*
Not disclosed	1038 (1.8)	<10 (<8.3)	4.14 (2.10–8.2)**	n/a	882 (1.6)	37 (5.7)	3.82 (2.74–5.35)**	1.72 (0.91–3.02)
**Educational attainment (key stage two)**^b^								
Lowest quintile	13,328 (24.4)	40 (33.3)	*reference*	*Reference*	10,586 (19.5)	174 (26.9)	*Reference*	*Reference*
Second	10,713 (19.6)	26 (21.6)	0.80 (0.40–1.32)	1.07 (0.60–1.90)	10,672 (19.6)	135 (20.9)	0.78 (0.62–0.97)*	1.01 (0.78–1.29)
Third	10,501 (19.2)	24 (20.0)	0.82 (0.49–1.36)	1.56 (0.87–2.78)	11,046 (20.3)	126 (19.5)	0.73 (0.58–0.92)**	1.18 (0.90–1.52)
Fourth	10,437 (19.1)	14 (11.7)	0.50 (0.27–0.92)*	1.01 (0.50–2.09)	10,974 (20.2)	127 (19.7)	0.77 (0.61–0.97)*	1.35 (1.04–1.77)*
Highest quintile	99689 (17.7)	16 (13.3)	0.73 (0.41–1.31)	1.75 (0.85–3.55)	11,112 (20.4)	84 (13.0)	0.55 (0.44–0.75)**	1.15 (0.85–1.57)
**Less than 80% attendance**^c^	2676 (4.9)	29 (24.2)	6.50 (4.24–9.92)**	3.50 (2.16–5.70)**	2430 (4.5)	130 (20.1)	5.42 (4.50–6.58)**	2.84 (2.70–3.51)**
**Fixed term exclusions**^a^	6054 (10.7)	32 (26.7)	2.88 (1.92–4.31)**	1.30 (0.78–2.15)	2696 (4.8)	120 (18.6)	4.41 (3.61–5.37)**	1.69 (1.32–2.15)**
**Other social factors**								
Summer birth (May-Aug)	19,615 (34.7)	47 (39·1)	1.21 (0.84–1.75)	1.23 (0.83–1.83)	19,104 (34.1)	222 (34·4)	1.02 (0.87–1.20)	1.02 (0.86–1.21)
Free school meals ^a^	13, 764 (24.4)	37 (30·8)	1.40 (0.95–2.05)	1.35 (0.87–2.10)	13,369 (22.1)	189 (29·3)	1.32 (1.11–1.56)**	1.22 (1.02–1.48)*
Looked after Child status ^d^	443 (0.8)	<10 (<8·3)	8.04 (3.75–17.3)**	3.18 (1.14–8.91)*	382 (0.7)	27 (4·3)	6.20 (4.22–9.12)**	3.16 (2.07–4.84)**
**ICD-10 Hyperkinetic disorder**	788 (1.4)	19 (15·8)	8.0 (5.0–12.8)**	4.36 (2.20–8.68)**	177 (0.3)	15 (1·8)	5.70 (3.42–9.50)**	3.58 (2.03–6.29)**

^*^P⩽0·05, ^**^P⩽0·01; missing values. ^a^257, ^b^3731, ^c^4049, ^d^4547, and ^e^Adjusted for all other factors listed in Tables 3 and 4

Eleven boys (0.5%) and fewer than 10 girls (<2.3%) with ASD presented with self-harm at a mean age of 15 (Table 2) [statistical disclosure rules prevent actual numbers being provided]. ASD was associated with nearly a three-fold increase in risk of self-harm in boys, showing little change after adjustment for a range of confounders (aHR 2·79, *P*<0·01); however, ASD was not a significant risk for girls.

In view of small numbers of individuals with both ASD and self-harm, we conducted an additional sensitivity analysis by performing penalised Cox regression [42]. Results were very similar to the main analysis for both boys: aHR 2.89 95% CI 1.39–5.45 and girls aHR 0.60, 95% CI 0.17–1.51. Full results for the penalised regressions can be found in Additional file 1: Table S1a and S1b.

### Association between other sociodemographic, clinical and educational factors and self-harm

Cox regression models (Tables 3 and 4) indicated, for both boys and girls, a strong inverse association between black ethnicity (relative to white ethnicity) and risk of presenting with self-harm. This association remained significant, and the effect estimate is consistent, after adjustment for a range of potential confounders. Asian ethnicity was associated with significantly reduced risks of self-harm presentation only amongst girls. English as a second language was associated with a decreased risk for both genders. Levels of neighbourhood deprivation were not found to be significantly associated with risk of self-harm. For girls specifically, being from a family eligible for free school meals was a significant predictive factor.

In terms of school-related factors, poor attendance at school was associated with self-harm for both boys and girls with and without ASD, and this association remained significant, although attenuated, after adjustment for confounders. For girls, those with the highest levels of academic attainment had a lower risk of self-harm in unadjusted analysis; however, after adjustment for confounders, being in the second from top academic quintile was associated with a higher risk. This association was not observed for boys. Finally, exclusion from school was associated with a higher risk of self-harm in both boys and girls, with the association remaining significant after adjustment for confounders in girls only.

Other significant predictors for self-harm in both genders included BES SEN, being a looked-after child and hyperkinetic disorder.

### Sensitivity analyses

Restricting the analyses to adolescents joining the study aged 11 showed that ASD in boys remained a significant risk factor (aHR 3·43, 95% CI 1·05–11.3, *p*<0·04). Restricting to those enrolled in mainstream school produced similar results (aHR 3·28, 95% CI 1·64–6·6, *p*<0·01). The final analyses used an imputed dataset, which replaced missing NPD variables that were either not supplied to DfE or missed matches between NPD and CRIS data. Additional file 2: Table S2 shows the distribution of key variables before and after multiple imputation, which was checked to establish the validity of this imputed dataset. Observed values of complete cases with imputed values showed similar distributions, with the exception of an increase in the proportion of adolescents who did not disclose their language status. Additional file 3: Table S3 shows fully adjusted effect estimates are similar to the complete case analyses (Tables 3 and 4) but with some gains in precision.

## Discussion

In this retrospective cohort study of 113,543 adolescents, we found that ASD was associated with nearly a three-fold increased risk of self-harm among boys. This association persisted after controlling for a range of potential confounders and was robust to multiple sensitivity analyses. Previous studies have reported an association between ASD and suicidal behaviours in adolescents; however, these have methodological weaknesses such as lack of appropriate comparison groups and have used measures of self-harm assessment such as parent report. These studies have also largely relied on clinical populations [22, 23] and therefore have not provided evidence that ASD is a risk factor for self-harm in a general population sample.

In addition to the association between ASD and self-harm, there were several other findings of note. Consistent with a number of studies examining socio-economic risk factors for self-harm [43, 44], free-school meal eligibility was significantly associated with self-harm in girls. The absence of an association between neighbourhood deprivation and self-harm is at odds with much previous research [45–47], however is consistent with recent findings that some deprived inner-city areas had paradoxically low rates of self-harm [48] possibly explained by complex social and behavioural factors in some communities [49]. Again consistent with previous evidence [50], we found looked after children were at a significant risk for self-harm..

In both boys and girls, there was an approximately three-fold increase in self-harm for those with persistent absence from school at baseline. As far as we are aware, this is the first population-based longitudinal study describing such an effect. These findings do not show that absenteeism causes self-harm but do suggest that is an important group to target for preventive interventions. Study findings of school exclusion predicting later self-harm were consistent with a small scale cross-sectional study showing significantly higher rates (22%) of self-harm amongst adolescents with a history of exclusion [51].

ADHD was a strong predictor of self-harm with approximately a four-fold increased risk for both genders, addressing a gap in the evidence with very few UK-based prospective studies exploring the association between ADHD and self-harm, particularly in girls [52]. Behavioural, emotional and social SEN were associated with a 3–4 times increased risk of self-harm on unadjusted analysis, which remained significant after adjusting for potential confounders. These results should be interpreted with some caution, as reverse causality could be driving these findings where self-harm, yet to present for emergency care, has led to allocation of this SEN status.

The fact that ASD was found to be a significant risk factor for boys only should not be taken to imply that boys with ASD are at a greater absolute risk of self-harm than girls with ASD. The absolute risk of self-harm amongst girls with ASD was similar to boys, and self-harm incidence rates in the whole population studied were far higher amongst girls (1.5% compared to 0.3% in boys). The gender discrepancy found in this study may be seen as inconsistent with recent findings suggesting adult women with ASD were 13 times more likely to die from suicide, compared to a 6-fold risk in males [53]. A possible explanation for this gender discrepancy is that several girls who self-harmed within this study may have had undiagnosed ASD and were included within general population rates, producing an underestimate of the true effect of ASD on self-harm due to misclassification. Under recognition and barriers to ASD diagnosis in girls has been more widely discussed within recent literature [54, 55].

### Strengths and limitations

Our use of routinely collected data from schools allowed for longitudinal follow-up on a very large population-based sample, with participation and retention of many individuals who would be at risk of attrition in traditional cohort designs [56]. The data linkage and extraction strategies using free text from electronic health records, provided detailed clinical information, reduced recall and observer bias and improved on the conventional health database studies of self-harm by detecting school-based risk factors. Consistent with other hospital-based studies, the rates presented are likely to represent a fraction of self-harm within the adolescent community. However, using free text extraction from mental health records at the time of presentation, consistent with previous research [57], far higher rates were found using this type of data in comparison to published figures of self-harm inpatient admission rates in HES.

However, our findings need to be interpreted in view of some limitations. The first is that although the overall sample used in the analysis is large, both the exposure (ASD) and the outcome (self-harm) were relatively rare. There is a potential risk of sparse-data bias [58] which could have resulted in inflated effect-size estimates. In view of this, we conducted a sensitivity analysis using Firth’s penalised regression approach which validated that our main findings were consistent following statistical mitigation.

In terms of other limitations, it is possible that those with ASD without associated learning difficulties are less likely to be identified and therefore will not receive a SEN ASD code. This may have led to an underestimation of the true association between ASD and self-harm and could mean that findings are less relevant for those with less severe ASD. Although we had information on learning difficulties which gave us some indication of ASD severity, we did not have specific ASD severity which is an important aspect to explore in future research. Additionally, due to the inability to capture exposure variables for the whole population at risk, biases may have arisen within the complete case analyses; however, subsequent analyses using imputed data were consistent, suggesting that possible biases did not significantly affect the study findings. As mentioned in the methods section, a potential source of bias when using linked data is that non-matching between datasets is more likely to be associated with certain characteristics, most often affecting disadvantaged groups [59]. Previous work using these data, found little impact of linkage bias on the association between education and health outcomes. The governance and methodological challenges of linking and analysing these health and educational data sources have been previously discussed in detail by Downs and colleagues [28]

It should also be noted that the data used for this study is for the years 2009–2013. Although the association between ASD and self-harm, as well as other factors included in this analysis, are not likely to vary significantly over relatively short periods of time, it is possible that any advances in diagnosis and management of ASD could have an impact on findings over time. However, the potential number of ‘missed’ ASD cases in our 2009–2013 cohort is likely to have a created an underestimate of the association between ASD and self-harm. No comparable recent data are currently available to explore this association beyond 2013; however, once such data do become available, further research could examine any changes over time.

In view of these strengths and limitations, there is much to be learned in order to develop and refine research methods using large, linked health and administrative datasets. We report a novel resource and methodology for answering important public health research questions in child and adolescent mental health and education. As we have highlighted, these data come with their own specific challenges, but the scale and richness of these linked data offer a wealth of opportunities to researchers and policy makers.

Our finding of an association between ASD and self-harm has several implications for policy and practice. The finding of an association before adjusting for confounders has importance from a policy perspective when identifying groups who may benefit from targeted self-harm prevention strategies. The fact that this association persists after adjusting for a range of confounders suggests that ASD itself may increase the risk of self-harm; however, the mechanism by which this occurs remains unclear. This may be explained by a combination of difficulties: delayed identification of needs, higher rates of psychopathology and a limited understanding of effective treatment targets for psychiatric co-morbidity in this group, relative to the general population [60, 61]. This however is largely theoretical, and further research into the mechanisms behind self-harm within adolescent ASD is warranted.

## Conclusions

To our knowledge, this is the first longitudinal investigation in a non-clinical population to examine the risk of hospital presentation with self-harm for adolescents with ASD. By using linked school census and routinely collected mental health data, this study included the whole population of four boroughs of South London, thus reducing the impact of selection bias often seen within traditional cohort studies. This study found that boys with ASD had a nearly three-fold increased risk of self-harm compared to boys without ASD. This association was not observed for girls. Several other clinical, social and educational factors were also found to be associated with increased risk including poor attendance at school and a history of being in foster care both associated with an approximately three-fold increase in risk, and exclusion from school with an approximately 50% increased risk. These findings are an important step in the development of strategies to prevent self-harm through the identification of vulnerable groups, but should be considered in light of the study’s limitations, particularly confounding variables that we were unable to adjust for. Further studies are needed to replicate these findings, including in other settings. This study also provides an example of how routinely collected public service data linkage can be used to tackle important public health issues and how large-scale epidemiological approaches to examining self-harm risk in adolescence can be enhanced.

## Supplementary Information

**Additional file 1: Table S1a**. An analysis of socio-demographic risk factors for emergency presentations with self-harm amongst 113, 543 adolescents residing in South London using crude and multivariable penalised cox-regression analyses. **Table S1b**. An analysis of educational and clinical risk factors for emergency presentations with self-harm (*n*=1020) amongst 113, 543 adolescents aged 11-17 residing in South London using crude and multivariable penalised cox-regression analyses.

**Additional File 2: Table S2**. The distribution of socio-demographic and educational variables before (original) and after multiple imputation.

**Additional File 3: Table S3**. An analysis of educational and clinical risks factors for emergency presentations at hospital with self-harm using multiple imputed data.

## Data Availability

Data are owned by a third-party South London and Maudsley Biomedical Research Centre Clinical Record Interactive Search tool that provides access to anonymised data derived from electronic medical records of the South London and Maudsley National Health Service Foundation Trust. These data can only be accessed by permitted individuals from within a secure firewall (i.e. remote access is not possible, and the data cannot be sent elsewhere) in the same manner as the authors.

## Abbreviations

ADHD: Attention deficit hyperactivity disorder
APC: Admitted patient care
ASD: Autism spectrum disorder
BES: Behavioural, emotional and social
CRIS: Clinical Record Interactive Search
DfE: Department for Education
ED: Emergency Department
ePJS: Electronic Patient Journey System
HES: Hospital episode statistics
aHR: Adjusted hazard ratio
KS2: Key stage 2
NHS: National Health Service
NICE: National Institute for Health and Care Excellence
NPD: National Pupil Database
SATs: Standardised Attainment Tests
SEN: Special Educational Needs
SLaM: South London and Maudsley NHS Foundation Trust
