# Supplementary material for: Autism spectrum disorders as a risk factor for adolescent self-harm: a retrospective cohort study of 113,286 young people in the UK
Source: BMC Med. 2022 Apr 29;20:137. doi: 10.1186/s12916-022-02329-w (PMC9052640; doi:10.1186/s12916-022-02329-w)
Supplement: Supplementary file 1 — Additional file 1: Table S1a. An analysis of socio-demographic risk factors for emergency presentations with self-harm amongst 113, 543 adolescents residing in South London using crude and multivariable penalised cox-regression analyses. Table S1b. An analysis of educational and clinical risk factors for emergency presentations with self-harm (n=1020) amongst 113, 543 adolescents aged 11-17 residing in South London using crude and multivariable penalised cox-regression analyses. [file 12916_2022_2329_MOESM1_ESM.docx]

*Table S1a:* An analysis of socio-demographic risk factors for emergency presentations with self-harm amongst 113, 543 adolescents residing in South London using crude and multivariable penalised cox-regression analyses.

|  | Male (n=56,648) | | |  | Female (n=56,897) | | |
| --- | --- | --- | --- | --- | --- | --- | --- |
| **Socio-demographic characteristics** | No self-harm (n=56,462) | Self-harm (n=186) | Adjusted Hazard Ratio |  | No self-harm (n=56,063) | Self-harm (n=834) | Adjusted Hazard Ratio |
| Mean age at baseline (SD) | 12·8 (2·1) | 14·1 (1·8) | 1·39 (1·22-1·57)** |  | 12·8 (2·0) | 13·9 (1·8) | 1·28 (1·22-1·35)** |
| Mean duration of follow-up (SD) | 2·73 (1·3) | 1·89 (1·2) | - |  | 2·70 (1·3) | 1·86 (1·1) | - |
| **Ethnicity** | (n, %) | (n, %) |  |  | (n, %) | (n, %) |  |
|  |  |  |  |  |  |  |  |
| White | 20,943 (37·1) | 88 (47·3) | *reference* |  | 20,534 (36·6) | 357(42·8) | *reference* |
| Black. | 20, 842 (36·9) | 28 (15·0) | 0·41 (0·24-0·68)** |  | 21,106 (37·7) | 212 (25·4) | 0·58 (0·47-0·71)** |
| Asian. | 4,860 (8·6) | 12 (6·5) | 0·97 (0·37-2·17) |  | 4,865 (8·7) | 47 (5·7) | 0·62 (0·40-0·93)* |
| Mixed | 6,234 (11·0) | 16 (8·6) | 0·75 (0·39-1·31) |  | 6,218 (11·1) | 102 (12·2) | 1·13 (0·89-1·42) |
| Other. | 1,968 (3·5) | 12 (6·5) | 0·81 (0·16-2·48) |  | 1,880 (3·3) | 29 (3·5) | 0·80 (0·46-1·29) |
| not disclosed. | 1,615 (2·9) | 30 (16·1) | 0·95 (0·20-2·80) |  | 1,460 (2·6) | 87 (10·4) | 0·96 (0·54-1·58) |
| **National neighbourhood deprivation ^a^** | |  |  |  |  |  |  |
| Most deprived quintile | 20,586 (36·5) | 63 (33·9) | *reference* |  | 20,144 (35·9) | 320 (38·4) | *reference* |
| 2nd | 22, 855 (40·5) | 78 (42·5) | 0·89 (0·49-1·61) |  | 22,720 (40·6) | 330 (39·5) | 1.46 (1.12-1.90)** |
| 3rd | 7,989 (14·2) | 32 (17·2) | 0.87 (0·47-1.60) |  | 8,193 (14·6) | 120 (14·4) | 1.14 (0.86-1.51) |
| 4th | 3,378 (6·0) | <10 (<5·4) | 0·98 (0·54-1·76) |  | 3,311 (5·9) | 44 (5·3) | 1.35 (1.03-1.77)* |
| Least deprived | 1,620 (2·9) | <10 (<5·4) | 0·84 (0·45-1·54) |  | 1,677 (3·0) | 20 (2·4) | 1.24 (0·95-1·64) |

* P⩽0·05 **P⩽0·01; missing values ^e^ Adjusted for all other factors listed in this table and supplementary table 4b

Supplementary table S1b: An analysis of educational and clinical risk factors for emergency presentations with self-harm (n=1020) amongst 113, 543 adolescents aged 11-17 residing in South London using crude and multivariable penalised cox-regression analyses.

|  | Male (n=56,581) | | |  | Female (n=56,709) | | |
| --- | --- | --- | --- | --- | --- | --- | --- |
| **Educational and clinical characteristics** | No self-harm (n=56,460) | Self-harm (n=120) | Adjusted Hazard Ratio |  | No self-harm (n=56,063) | Self-harm (n=646) | Adjusted Hazard Ratio |
|  | (n, %) | (n, %) |  |  | (n, %) | (n, %) |  |
| **Special Education Needs ^a^** |  |  |  |  |  |  |  |
| Autism Spectrum Disorders | 2,015 (3·5) | 11 (9·2) | 2.89 ((1.39-5.45)** |  | 434 (0·8) | <10 (<1·5) | 0·60 (0·17-1·51) |
| Learning Difficulties (specific/moderate) | 9,418 (16·7) | 28 (23·3) | 1·09 (0·65-1.77) |  | 6,113 (10·9) | 105 (16·3) | 1.00 (0·77-·1.29) |
| Learning Difficulties (severe/profound) | 840 (1·5) | <10 (<8·3) | 0·59 (0·07-2·26) |  | 444 (0·8) | <10 (<1·5) | 0·50 (0·10-1·43) |
| Behavioural, Emotional, Social | 7,235 (12·8) | 39 (32·5) | 1·68 (1.01-2·74)* |  | 3,494 (6·2) | 143 (22·1) | 2·31 (1·84-2·89)** |
| Speech, language and communication | 5,086 (9·0) | 11 (9·2) | 1.04 (0·51-1·95) |  | 2,269 (4·1) | 26 (4·0) | 1·15 (0·74-1·70) |
| Hearing, vision or physical disability | 860 (1·5) | <10 (<8·3) | 2·41 (0·79-5·60) |  | 746 (1·3) | 5 (0·8) | 0·66 (0·25-1·38) |
| **First language ^a^** |  |  |  |  |  |  |  |
| English | 41,482 (73·5) | 100 (83·3) | *reference* |  | 40,652 (72·5) | 508 (78·6) | *reference* |
| Other. | 13,942 (24·7) | 11 (9·2) | 0·52 (0·25-0·99)* |  | 14, 529 (25·9) | 101 (15·6) | 0·77 (0·61-0·98)* |
| Not disclosed. | 1,038 (1·8) | <10 (<8·3) | NA |  | 882 (1·6) | 37 (5·7) | 1·78 (0·97-2.99) |
| **Educational attainment (Key stage two)** ^b^ | |  |  |  |  |  |  |
| Lowest quintile | 13,328 (24·4) | 40 (33·3) | *reference* |  | 10,586 (19·5) | 174 (26·9) | *reference* |
| Second. | 10,713 (19·6) | 26 (21·6) | 1·07 (0·59-1·88) |  | 10,672 (19·6) | 135 (20·9) | 1·01 (0·79-1·30) |
| Third. | 10,501 (19·2) | 24 (20·0) | 1·54 (0·85-2·74) |  | 11,046 (20·3) | 126 (19·5) | 1·18 (0·91-1·53) |
| Fourth. | 10,437 (19·1) | 14 (11·7) | 1.00 (0·49-1.98) |  | 10,974 (20·2) | 127 (19·7) | 1·36 (1·04-1·78)* |
| highest quintile. | 9,9689 (17·7) | 16 (13·3) | 1.71 (0·83-3.42) |  | 11,112 (20·4) | 84 (13·0) | 1·16 (0·85-1·57) |
| **Less than 80% attendance ^c^** | 2,676 (4·9) | 29 (24·2) | 3.59 (2·19-5.74)** |  | 2,430 (4·5) | 130 (20·1) | 2·83 (2·27-3·51)** |
| **Fixed term exclusions** ^a^ | 6,054 (10·7) | 32 (26·7) | 1·32 (0·78-2·17) |  | 2696 (4·8) | 120 (18·6) | 1·68 (1·31-2·14)** |
| **Other social factors** |  |  |  |  |  |  |  |
| Summer birth (May-Aug) | 19,615 (34·7) | 47 (39·1) | 1·23 (0·83-1·82) |  | 19,104 (34·1) | 222 (34·4) | 1·02 (0·86-1·20) |
| Free school meals ^a^ | 13, 764 (24·4) | 37 (30·8) | 1·39 (0·89-2.14) |  | 13,369 (22·1) | 189 (29·3) | 1·22 (1·02-1·47)* |
| Looked after Child status ^d^ | 443 (0·8) | <10 (<8·3) | 3·48 (1·12-8·31)* |  | 382 (0·7) | 27 (4·3) | 3·26 (2·08-4·87)** |
| **ICD-10 Hyperkinetic disorder** | 788 (1·4) | 19 (15·8) | 4.59 (2.23-8.72)** |  | 177 (0·3) | 15 (1·8) | 3·68 (2·01-6·17)** |

* P⩽0·05 **P⩽0·01; missing values ^e^ Adjusted for all other factors listed in this table and supplementary table 4a
