# Supplementary material for: Autism spectrum disorders as a risk factor for adolescent self-harm: a retrospective cohort study of 113,286 young people in the UK
Source: BMC Med. 2022 Apr 29;20:137. doi: 10.1186/s12916-022-02329-w (PMC9052640; doi:10.1186/s12916-022-02329-w)
Supplement: Supplementary file 2 — Additional File 2: Table S2. The distribution of socio-demographic and educational variables before (original) and after multiple imputation. [file 12916_2022_2329_MOESM2_ESM.docx]

Table S2: The distribution of socio-demographic and educational variables before (original) and after multiple imputation.

| Socio-demographic and clinical characteristics |  | Male (n=56,648) | | | | |  | Female (n=56, 709) | | | | |  |
| --- | --- | --- | --- | --- | --- | --- | --- | --- | --- | --- | --- | --- | --- |
|  |  | Original % | |  | Imputed % | |  | Original % | |  | Imputed % | | |
|  |  | No self-harm | Self-harm |  | No self-harm | Self-harm |  | No self-harm | Self-harm |  | No self-harm | Self-harm | |
| **National neighbourhood deprivation** |  |  |  |  |  |  |  |  |  |  |  |  | |
| Most deprived quintile |  | 36·5 | 33·9 |  | 33·6 | 37·5 |  | 35·9 | 38·4 |  | 32·8 | 37·5 | |
| 2nd |  | 40·5 | 42·5 |  | 40·8 | 38·1 |  | 40·8 | 39·5 |  | 40·6 | 38·1 | |
| 3rd |  | 14·2 | 17·2 |  | 15·4 | 16·3 |  | 14·6 | 14·4 |  | 16·3 | 17·2 | |
| 4th |  | 6·0 | 4·4 |  | 7·0 | 7·2 |  | 5·9 | 5·3 |  | 7·2 | 5·4 | |
| Least deprived quintile |  | 2·9 | 2·2 |  | 3·2 | 3·3 |  | 3·0 | 2·4 |  | 3·3 | 2·2 | |
| **Special Education Needs ^a^** |  |  |  |  |  |  |  |  |  |  |  |  | |
| Autism Spectrum Disorders |  | 3·5 | 9·2 |  | 3·5 | 5·1 |  | 0·8 | 0·5 |  | 0·7 | 0·8 | |
| Learning Difficulties (specific/moderate) |  | 16·7 | 23·3 |  | 14·4 | 24·4 |  | 10·9 | 16·3 |  | 9·3 | 18·5 | |
| Learning Difficulties (severe/profound) |  | 1·5 | 0·8 |  | 1·4 | 1·0 |  | 0·8 | 0·3 |  | 0·7 | 0·4 | |
| Behavioural, Emotional, Social problems |  | 12·8 | 33·5 |  | 12·5 | 41·4 |  | 6·2 | 22·1 |  | 6·0 | 23·2 | |
| Speech, language and communication |  | 9·0 | 9·2 |  | 7·5 | 8·3 |  | 4·1 | 4·0 |  | 3·4 | 3·9 | |
| Hearing, vision or physical disability |  | 1·5 | 3·3 |  | 1·3 | 1·6 |  | 1·3 | 0·8 |  | 1·3 | 0·8 | |
| **First language ^a^** |  |  |  |  |  |  |  |  |  |  |  |  | |
| English |  | 73·5 | 83·3 |  | 72·0 | 66·5 |  | 72·5 | 78·6 |  | 72·1 | 68·8 | |
| Other |  | 24·7 | 9·2 |  | 23·0 | 11·3 |  | 25·9 | 15·6 |  | 25·6 | 13·8 | |
| Not disclosed |  | 1·8 | 7·5 |  | 5·1 | 24·2 |  | 1·6 | 5·7 |  | 4·3 | 17·5 | |
| **Educational attainment (Key stage two) ^b^** |  |  |  |  |  |  |  |  |  |  |  |  | |
| Lowest quintile |  | 24·4 | 33·0 |  | 23·2 | 39·6 |  | 19·5 | 27·0 |  | 17·7 | 29·6 | |
| second |  | 19·6 | 21·2 |  | 18·0 | 20·0 |  | 19·6 | 20·9 |  | 18·3 | 21·6 | |
| third |  | 19·2 | 20·3 |  | 18·5 | 14·3 |  | 20·3 | 19·4 |  | 19·1 | 17·7 | |
| fourth |  | 19·1 | 11·9 |  | 19·9 | 13·7 |  | 20·2 | 19·7 |  | 21·3 | 16·9 | |
| highest quintile |  | 17·7 | 13·6 |  | 20·5 | 12·4 |  | 20·4 | 13·0 |  | 23·6 | 14·3 | |
| **Less than 80% attendance ^c^** |  | 4·9 | 26·4 |  | 5·5 | 31·3 |  | 4·5 | 21·2 |  | 5·0 | 25·7 | |
| **Fixed term exclusions ^a^** |  | 10·7 | 26·7 |  | 10·9 | 37·8 |  | 4·8 | 18·6 |  | 5·3 | 23·2 | |
| **Other social factors** |  |  |  |  |  |  |  |  |  |  |  |  | |
| Summer birth (May -Aug) |  | 34·4 | 41·6 |  | 34·7 | 36·3 |  | 34·1 | 34·4 |  | 33·8 | 34·5 | |
| Free school meals ^a^ |  | 24·4 | 30·8 |  | 21·9 | 32·5 |  | 22·1 | 29·3 |  | 20·1 | 30·2 | |
