# Supplementary material for: Autism spectrum disorders as a risk factor for adolescent self-harm: a retrospective cohort study of 113,286 young people in the UK
Source: BMC Med. 2022 Apr 29;20:137. doi: 10.1186/s12916-022-02329-w (PMC9052640; doi:10.1186/s12916-022-02329-w)
Supplement: Supplementary file 3 — Additional File 3: Table S3. An analysis of educational and clinical risks factors for emergency presentations at hospital with self-harm using multiple imputed data. [file 12916_2022_2329_MOESM3_ESM.docx]

Table S3: An analysis of educational and clinical risks factors for emergency presentations at hospital with self-harm using multiple imputed data.

|  | Imputed Sample | | |
| --- | --- | --- | --- |
| **Socio-demographic, educational and clinical characteristics** | Male |  | Female |
|  | Adjusted Hazard Ratio |  | Adjusted Hazard Ratio |
|  |  |  |  |
| **Mean age at baseline (SD)** | 1·63 (1·47-1·80)** |  | 1·36 (1·31-1·42)** |
|  |  |  |  |
| **Ethnicity** |  |  |  |
| White | *reference* |  | *reference* |
| Black | 0·38 (0·24-0·58)** |  | 0·60 (0·50-0·72)** |
| Asian | 1·23 (0·63-2·39) |  | 0·87 (0·63-1·20) |
| Mixed | 0·61 (0·35-1·04) |  | 0·95 (0·76-1·18) |
| Other | 2·60 (1·34-5·01)** |  | 1·18 (0·79-1·77) |
| not disclosed / unknown | 2·89 (0·17-3·04)** |  | 2·21 (1·57-3·10) |
| **National neighbourhood deprivation ^a^** |  |  |  |
| Most deprived quintile | *reference* |  | *reference* |
| 2nd | 1·17(0·85-1·62) |  | 0·96 (0·82-1·12) |
| 3rd | 1·32 (0·85-2·06) |  | 0·98 (0·79-1·23) |
| 4th | 0·76 (0·35-1·62) |  | 0·83 (0·60-1·15) |
| Least deprived quintile | 0·76 (0·27-2·07) |  | 0·76 (0·48-1·21) |
| **Special Education Needs ^a^** |  |  |  |
| Autism Spectrum Disorders | 2·32 (1·28-4·26**) |  | 0·62 (0·16-1·63) |
| Learning Difficulties (specific/moderate) | 1·16 (0·83-1·89) |  | 1·11 (0·88-1·39) |
| Learning Difficulties (severe/profound) | 0·41 (0·05-3·14) |  | 0·44 (0·11-1·77) |
| Behavioural, Emotional, Social problems | 2·02 (1·27-3·22)** |  | 2·08 (1·67-2·58)** |
| Speech, language and communication | 1·07 (0·53-2·15) |  | 1·16 (0·77-1·74) |
| Hearing, vision or physical disability | 1·58 (0·60-4·15) |  | 0·57 (0·23-1·41) |
| **First language** |  |  |  |
| English | *reference* |  | *reference* |
| Other | 0·48 (0·25-0·85)** |  | 0·70 (0·55-0·89)** |
| Not disclosed | 0·98 (0·33-2·86) |  | 1·37 (0·93-2·05) |
| **Educational attainment (Key stage two)** |  |  |  |
| Lowest quintile | *reference* |  | *reference* |
| second | 1·09 (0·70-1·69) |  | 1·05 (0·80-1·37) |
| third | 1·33 (0·76-2·43) |  | 1·18 (0·93-1·51) |
| fourth | 1·11 (0·58-2·13) |  | 1·32 (0·99-1·74) |
| highest quintile | 1·76 (0·96-3·23) |  | 1·17 (0·87-1·57) |
| **Less than 80% attendance ^c^** | 3·03 (1·87-5·01)** |  | 2·67 (2·18 -3·26)** |
| **Fixed term exclusions** | 1·25 (0·83-1·89) |  | 1·60 (1·26-2·01)** |
| **Other social factors** |  |  |  |
| Summer birth (May -Aug) | 1·20 (0·83-1·72) |  | 1·01 (0·86-1·19) |
| Free school meals ^a^ | 1·30 (0·85-1·97) |  | 1·27 (1·07-1·50)** |
| Looked after Child status ^d^ | 3·71 (1·88-7·35)** |  | 2·78 (1·94-3·97)** |
| ICD-10 Hyperkinetic disorder | 3·96 (2·27-6·93)** |  | 3·22 (1·91-5·43)** |
| **P<0·05, **P<0·01* |  |  |  |
